# Supplementary material for: Effects of Gryllus bimaculatus and Oxya chinensis sinuosa extracts on brain damage via blood-brain barrier control and apoptosis in mice with pentylenetetrazol-induced epilepsy
Source: PLoS One. 2023 Sep 11;18(9):e0291191. doi: 10.1371/journal.pone.0291191 (PMC10495007; doi:10.1371/journal.pone.0291191)

Fig 3. Tight junction levels

Claudin-5 expression levels

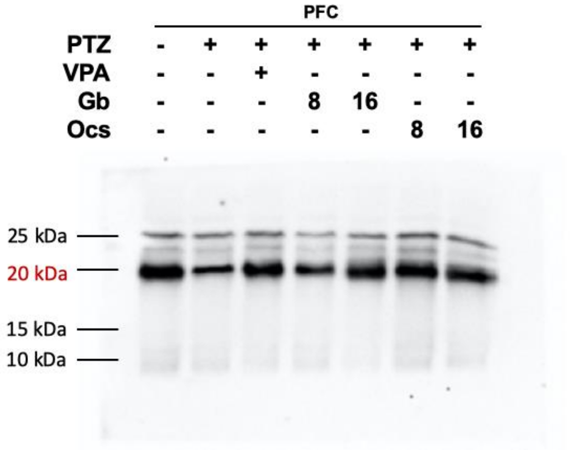

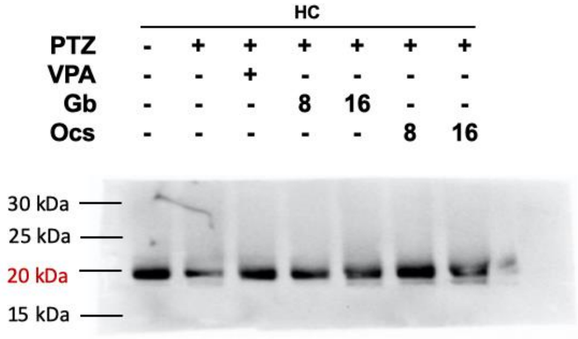

Occludin expression levels

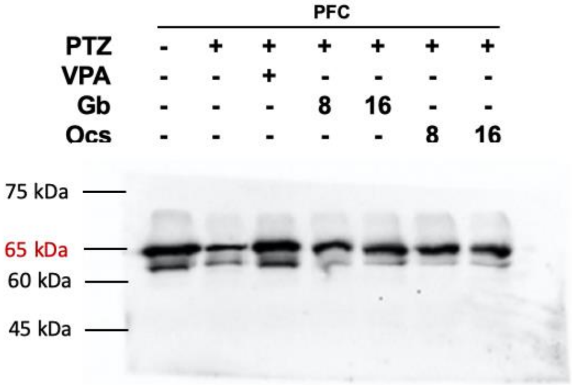

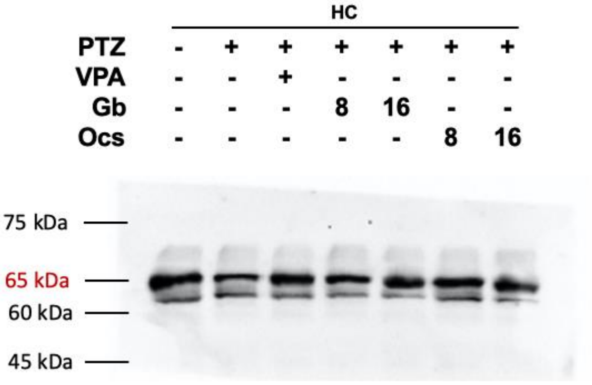

ZO-1 expression levels

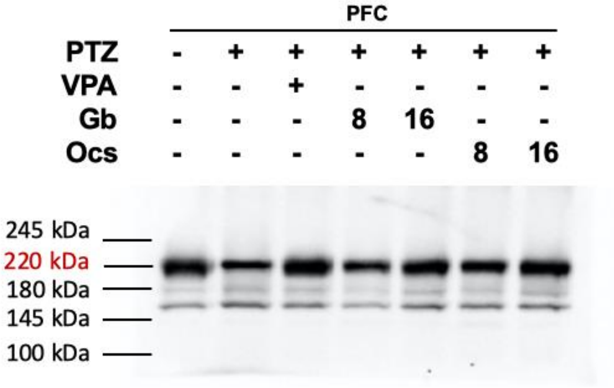

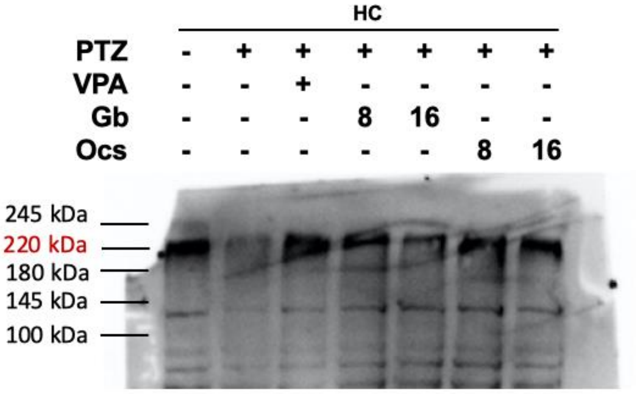

MMP-2 expression levels

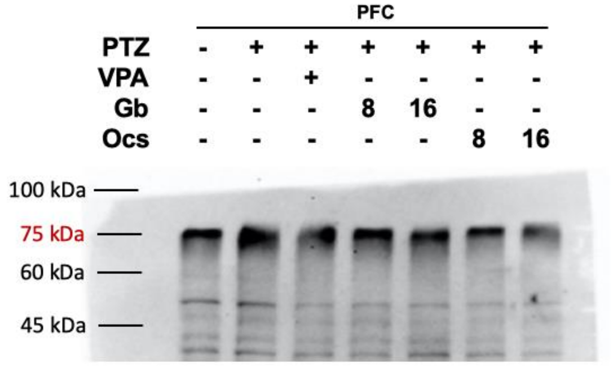

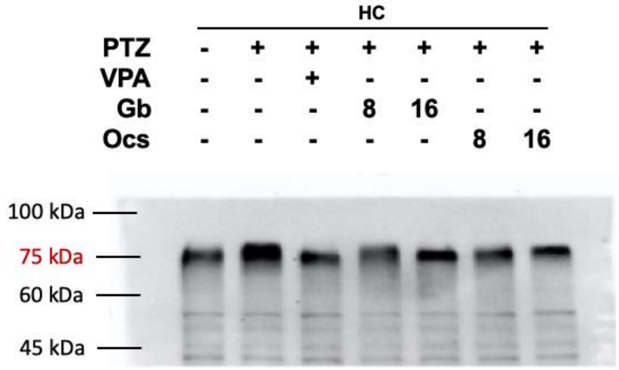

Fig 4. Acetylcholine activity and neuronal damage levels

AChE expression levels

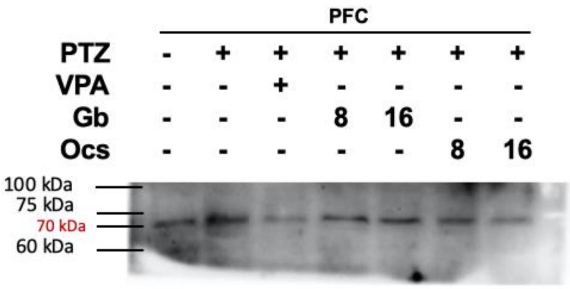

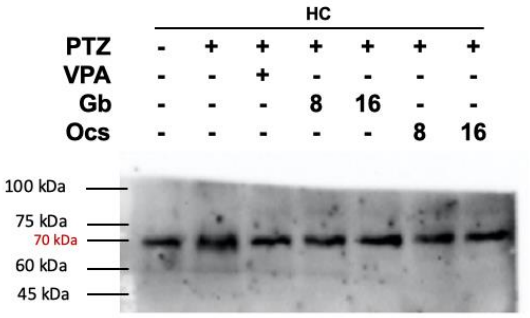

ChAT expression levels

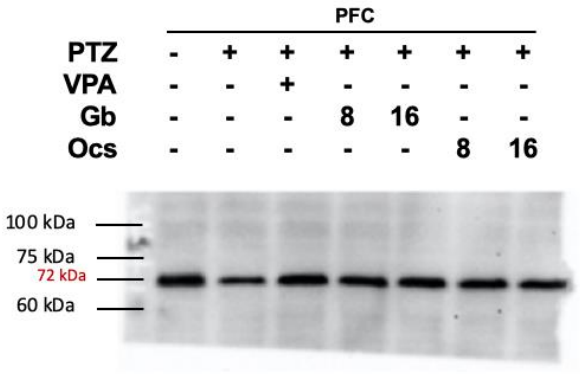

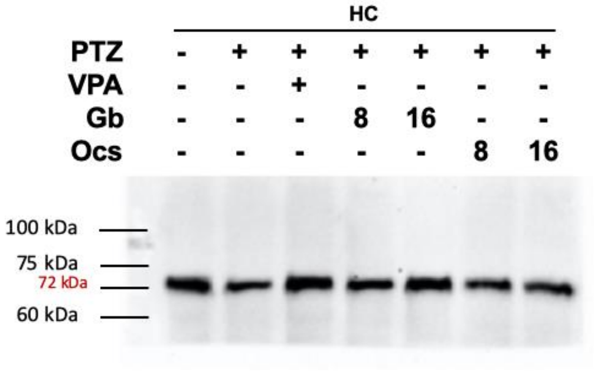

PSD-95 expression levels

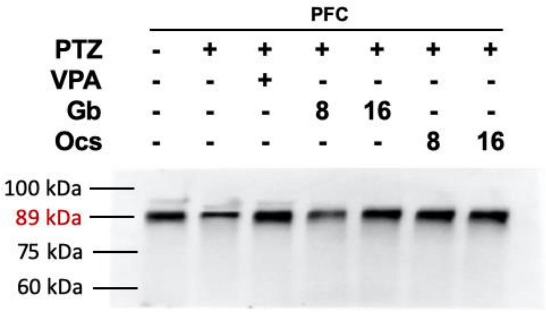

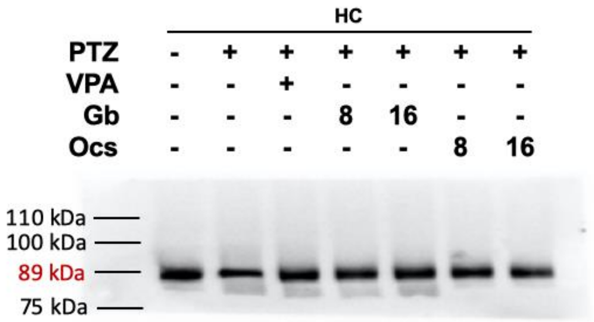

Neu-N expression levels

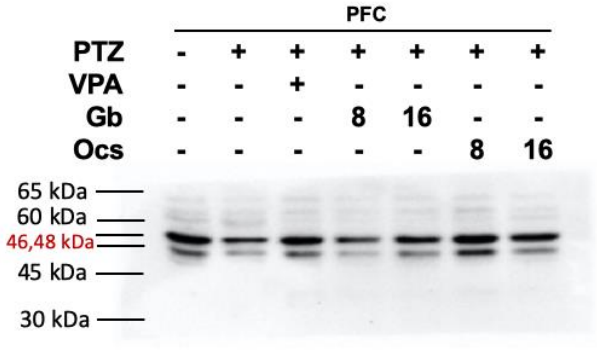

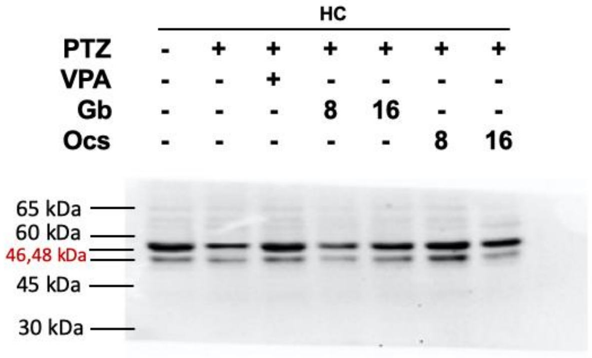

Fig 5. The excitatory and inhibitory neurotransmitter receptor-associated epileptogenesis

GRM2/3 expression levels

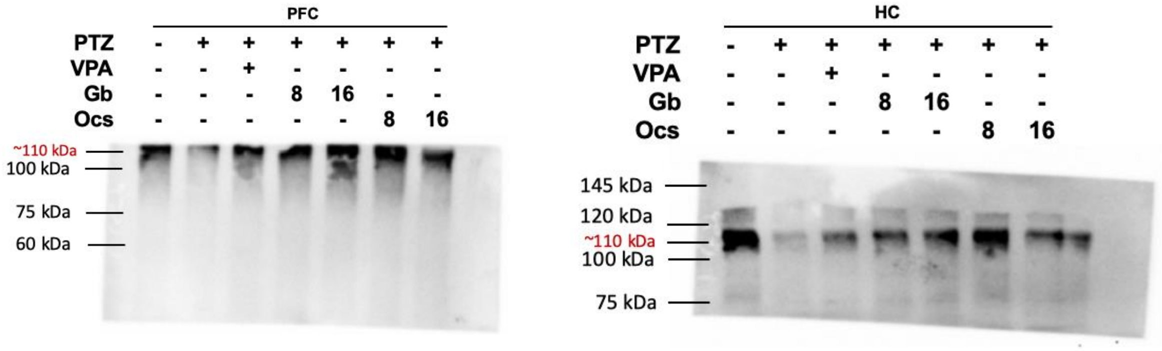

GAT1 expression levels

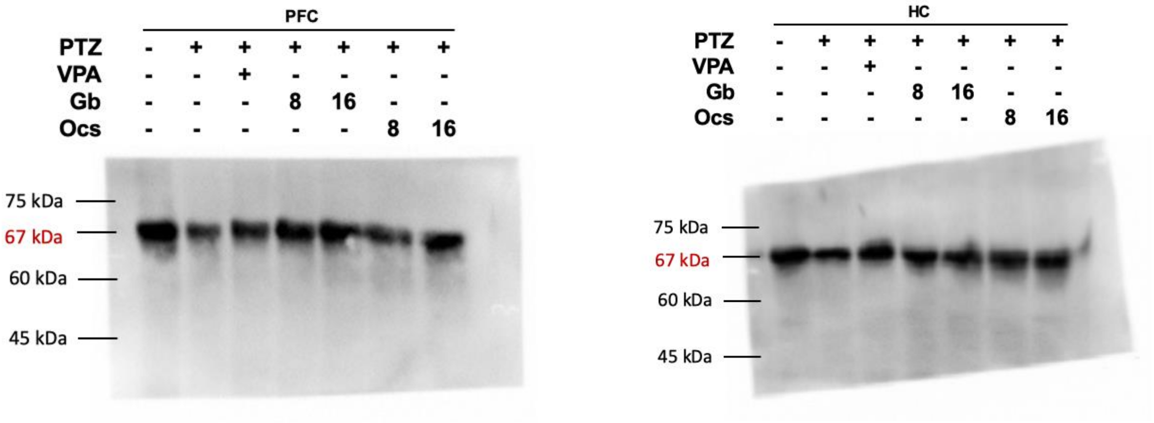

BDNF expression levels

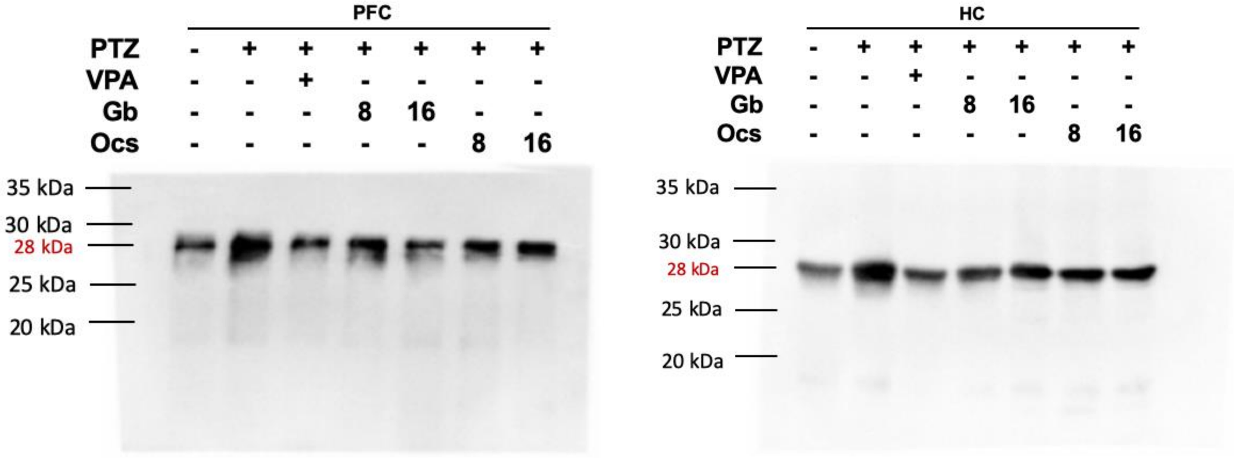

Fig 7. Apoptosis levels

Bcl-XL expression levels

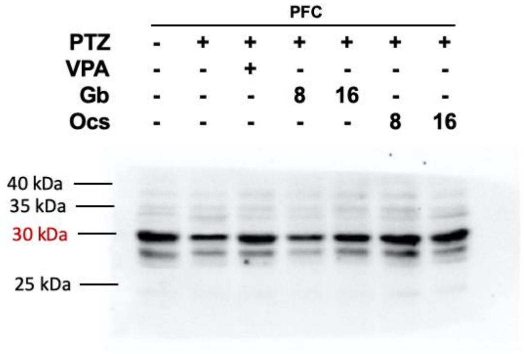

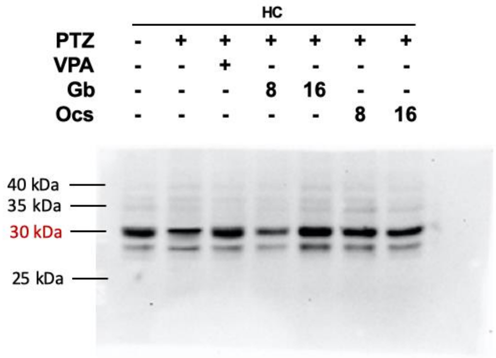

Bax expression levels

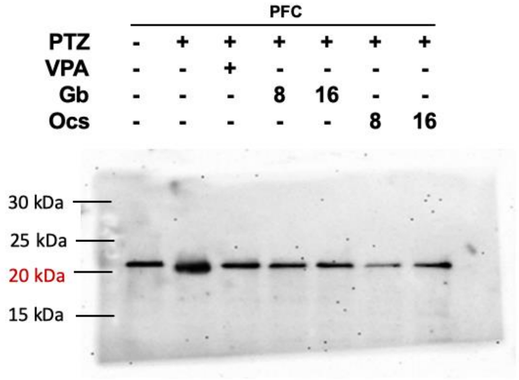

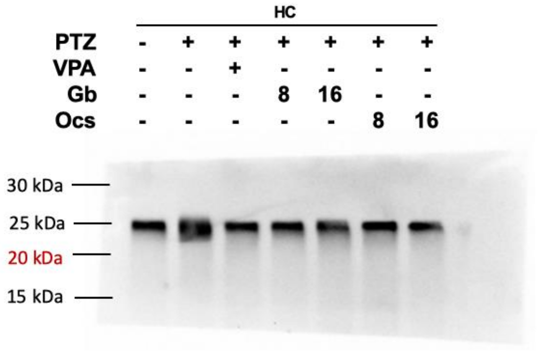

c-PARP expression levels

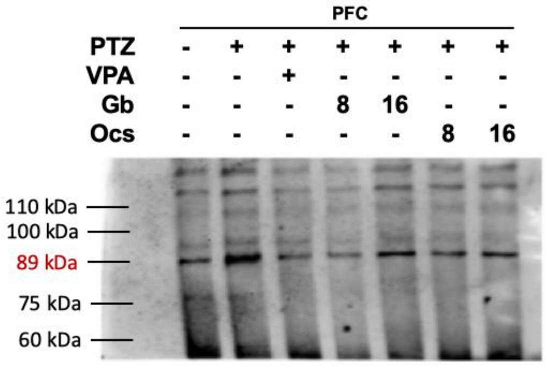

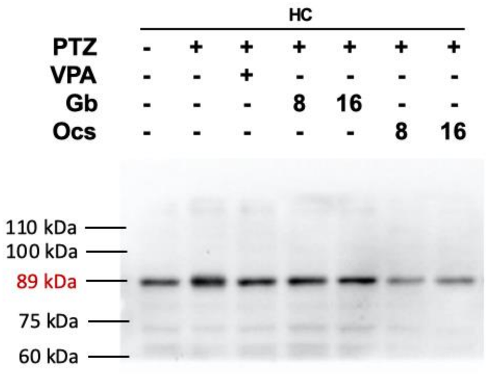

c-Caspase-3 expression levels

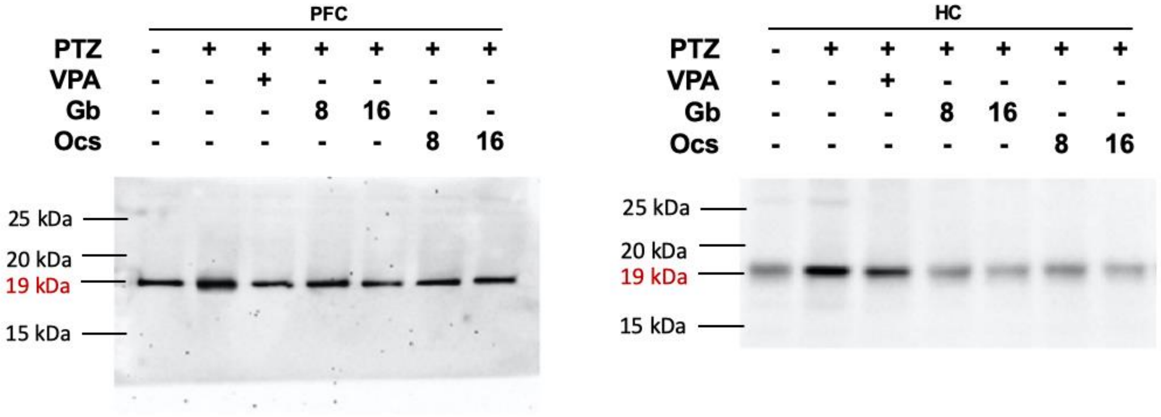

Supplement: S1 File — (PDF) [file pone.0291191.s001.pdf]
