# Supplementary material for: Effects of Gryllus bimaculatus and Oxya chinensis sinuosa extracts on brain damage via blood-brain barrier control and apoptosis in mice with pentylenetetrazol-induced epilepsy
Source: PLoS One. 2023 Sep 11;18(9):e0291191. doi: 10.1371/journal.pone.0291191 (PMC10495007; doi:10.1371/journal.pone.0291191)
Supplement: S1 Table — (DOCX) [file pone.0291191.s002.docx]

**SUPPLEMENTARY DATA**

**Supplementary Table S1.** Percent mortality of mice used in PTZ-dose optimization

|  | **PTZ** | | | | |
| --- | --- | --- | --- | --- | --- |
|  | **0 mg/kg** | **20 mg/kg** | **40 mg/kg** | **60 mg/kg** | **80 mg/kg** |
| **Before test (n*)** | 8 | 8 | 8 | 8 | 8 |
| **After test (n*)** | 8 | 8 | 8 | 6 | 4 |
| **% Mortality** | 0 | 0 | 0 | 25 | 50 |

n*: number of mice
