# Supplementary material for: Effects of Gryllus bimaculatus and Oxya chinensis sinuosa extracts on brain damage via blood-brain barrier control and apoptosis in mice with pentylenetetrazol-induced epilepsy
Source: PLoS One. 2023 Sep 11;18(9):e0291191. doi: 10.1371/journal.pone.0291191 (PMC10495007; doi:10.1371/journal.pone.0291191)
Supplement: S2 Table — (DOCX) [file pone.0291191.s003.docx]

**SUPPLEMENTARY DATA**

**Supplementary Table S2.** Percent mortality of mice used in the pilot study for optimization of insect extract concentrations

|  |  | **PTZ, 40 mg/kg** | | | | | |
| --- | --- | --- | --- | --- | --- | --- | --- |
|  | **CTL** | **Vehicle** | **VPA** | **Gb8** | **Gb16** | **Ocs8** | **Ocs16** |
| **Before test (n*)** | 10 | 10 | 10 | 10 | 10 | 10 | 10 |
| **After test (n*)** | 10 | 10 | 10 | 10 | 10 | 10 | 10 |
| **% Mortality** | 0 | 0 | 0 | 0 | 0 | 0 | 0 |

n*: number of mice; VPA, 100 mg/kg; Gb8, 8 g/kg; Gb16, 16 g/kg; Ocs8, 8 g/kg;

Ocs16, 16 g/kg; Vehicle, PTZ 40 mg/kg alone
