# Supplementary material for: Effects of Gryllus bimaculatus and Oxya chinensis sinuosa extracts on brain damage via blood-brain barrier control and apoptosis in mice with pentylenetetrazol-induced epilepsy
Source: PLoS One. 2023 Sep 11;18(9):e0291191. doi: 10.1371/journal.pone.0291191 (PMC10495007; doi:10.1371/journal.pone.0291191)
Supplement: S3 Table — (DOCX) [file pone.0291191.s004.docx]

**SUPPLEMENTARY DATA**

**Supplementary Table S3.** Records of the onset of seizures with different concentrations of the insect extracts

n*: number of mice; VPA, 100 mg/kg; Gb8, 8 g/kg; Gb16, 16 g/kg; Gb20, 20 g/kg; Ocs8, 8 g/kg;

Ocs16, 16 g/kg; Ocs20, 20 g/kg; Vehicle, PTZ 40 mg/kg alone

|  |  | **PTZ, 40 mg/kg** | | | | | | | |
| --- | --- | --- | --- | --- | --- | --- | --- | --- | --- |
|  | **CTL** | **Vehicle** | **VPA** | **Gb8** | **Gb16** | **Gb20** | **Ocs8** | **Ocs16** | **Ocs20** |
| **Number of mice** | 8 | 8 | 8 | 8 | 8 | 8 | 8 | 8 | 8 |
| **Seizure onset time (s)** | 0 | **24.5** | 0 | 62 | 79.8 | 73.2 | 76.8 | 102.7 | 91 |
| **Total duration of seizures (m)** | 0 | **10.3** | 1.3 | 4.2 | 2.5 | 3.1 | 4.0 | 1.6 | 2.4 |
| **% Mortality** | 0 | 0 | 0 | 0 | 0 | **33.3** | 0 | 0 | **16.7** |
| **Evaluated** | No seizure  symptoms | Early onset of seizures/ longer seizure duration | No seizure symptoms | Im  provement of seizure symptom | | | | | |
